# The Early Age Hydration Products and Mechanical Properties of Autoclaved Cement Paste Incorporating Supplementary Cementitious Materials

**DOI:** 10.3390/gels12020160

**Published:** 2026-02-12

**Authors:** Baoliang Li, Sahi Wail, Liying Shi, Arifuggaman Arif, Binbin Huo, Yongzhen Cheng

**Affiliations:** 1 Faculty of Architecture and Civil Engineering, Huaiyin Institute of Technology, Huaian 223001, China; lbljndx@126.com (B.L.); liawisah@gmail.com (S.W.); sly18151607639@163.com (L.S.); arifcumt96@gmail.com (A.A.); 2 Jiangsu Key Laboratory of Construction Materials, School of Materials Science and Engineering, Southeast University, Nanjing 211189, China; huobinbin@cumt.edu.cn; 3 School of Mines, China University of Mining and Technology, Xuzhou 221116, China

**Keywords:** autoclave curing, GBFS, FNS, LS, SS, hydration products, mechanical properties

## Abstract

This study systematically investigated the effects of four supplementary cementitious materials (SCMs), namely ferronickel slag (FNS), lithium slag (LS), steel slag (SS), and ground granulated blast furnace slag (GBFS), on various properties of autoclaved cementitious materials. Cement pastes and mortars with 0% and 30% replacement levels were prepared to examine their impacts on early age hydration products and mechanical properties, with comparisons made to specimens under standard 28-day curing. Key findings reveal that autoclaving promoted the formation of tobermorite, crystalline calcium aluminosilicate hydrate (CASH), gypsum and hydrogarnet, with the latter two phases potentially compromising concrete durability. Autoclave curing significantly enhanced SCM reactivity, as evidenced by thermogravimetric analysis: the mass loss below 200 °C (mainly from C–S–H gels decomposition) in SCM-incorporated pastes ranged from 87.0% (SS) to 104.6% (GBFS) of the control value, while the portlandite (Ca(OH)_2_) content decreased to between 47.7% (LS) and 82.4% (GBFS) of the control. Among the SCMs studied, autoclaving exhibited the most pronounced activation effect on LS, which also showed superior potential as a GBFS alternative in autoclaved concrete products. However, the low CaO content and acidic nature of LS limited its use to low replacement levels unless supplementary sources of alkalinity and CaO were provided.

## 1. Introduction

Autoclave curing is a specialized method that accelerates the cement hydration process through the application of elevated temperature and pressure [1]. Currently, it is extensively utilized in manufacturing ultra-high performance concrete (UHPC), reactive powder concrete [2], prestressed high-strength concrete pipe piles [3], aerated concrete [4], and other construction materials. This method enables materials with limited reactivity at ambient temperature to undergo thorough chemical reactions. For instance, it has been established that the dissolution of surface compounds of quartz sand during autoclave curing enhances the interfacial bond between the aggregate and the cement paste [5]. Moreover, autoclaving can effectively accelerate the reactions of components with low soundness characteristics [6]. Typical examples of such components include free calcium oxide (*f*-CaO) and magnesium-bearing phases such as free magnesium oxide (*f*-MgO). Consequently, autoclave curing is often employed as a method to evaluate the soundness of cement-based materials [7]. Additionally, autoclaving can mitigate the drawbacks associated with supplementary cementitious materials (SCMs) in concrete, such as prolonged setting times and low early-age strength [1]. When properly implemented, autoclave curing plays a crucial role in enabling concrete components to rapidly attain target strength levels, minimize shrinkage, and enhance resistance to various forms of erosion [8]. This approach also reduces cement consumption and the storage space required for precast product inventories, thereby offering significant economic and practical benefits to the construction industry.

Autoclave curing significantly alters the phase composition and microstructural characteristics of cement hydration products. During autoclave curing, the temperature typically ranges from 90 to 250 °C, and the pressure varies from 0.1 to 4.0 MPa [9]. In this environment, ettringite (AFt) decomposes and transforms into phases such as Monosulfoaluminate hydrate (AFm) in the initial stage. Simultaneously, autoclave curing can significantly increase the content of high-density calcium silicate hydrate (C−S−H) with a high elastic modulus in the matrix, which exhibits excellent mechanical properties [10]. Furthermore, as the curing temperature increases, crystalline phases including tobermorite and xonotlite form. These phases are characterized by a lower Ca/Si ratio, a higher average chain length, and a greater degree of polymerization, and their formation exerts a highly positive effect on enhancing the mechanical properties of concrete [2,11].

Nevertheless, autoclaving also introduces several long-term issues that require careful consideration. This process promotes the formation of hydrogarnet and delayed ettringite formation (DEF), both of which are known to compromise long-term mechanical performance and dimensional stability [12,13]. Additionally, the amorphous C−S−H gels formed under ambient temperature conditions typically exhibit a highly developed specific surface area and strong adhesion to aggregates. However, this adhesion is weakened by the increased crystallinity of C−S−H gels under autoclave curing conditions [14]. Meanwhile, the transformation of amorphous C−S−H gels into crystalline phases such as α-C_2_SH or truscotite during autoclave curing can also lead to the deterioration of concrete’s mechanical properties [15]. However, this issue can be mitigated by incorporating silicon sources such as quartz powder, which facilitates the transformation of α-C_2_SH into tobermorite [11]. Moreover, autoclave curing increases the capillary porosity of cement-based materials while reducing gels porosity, which further results in decreased mechanical properties and durability [3,12,16,17,18].

Studies have shown that incorporating SCMs can mitigate internal damage, such as cracking, in concrete subjected to autoclave curing. For example, silica fume can enhance the mechanical properties of autoclaved reactive powder concrete, and it can be partially replaced by other silicon sources such as ground granulated blast furnace slag (GBFS) and fly ash [2,11,12]. The addition of fly ash not only alleviates the reduction in concrete’s fracture toughness but also significantly decreases chloride ion permeability in autoclaved concrete [17,19]. In addition, SCMs can effectively inhibit the alkali-silica reaction (ASR) in concrete [20,21,22,23,24]. For instance, via pozzolanic reactions, SCMs such as silica fume, fly ash, and GBFS can mitigate ASR by consuming calcium hydroxide (CH), lowering the pore solution pH, and generating additional C−S−H gels [20]. Among these admixtures, silica fume is recognized as one of the most effective SCMs for ASR inhibition [23,24], owing to its high content of highly reactive silica and ultrafine particle size, which impart excellent pozzolanic activity. This allows silica fumes to rapidly consume CH, drastically reduce pore solution alkalinity, and significantly refine the concrete pore structure. Furthermore, Al-rich SCMs can enhance the alkali ion adsorption capacity of C−S−H gels, thereby reducing the alkali concentration in the pore solution and effectively suppressing ASR-induced concrete expansion [21,22]. It should also be noted that a higher CaO/SiO_2_ ratio in SCMs reduces their effectiveness in suppressing ASR, which is particularly evident in the comparison between Class C and Class F fly ashes, with the latter exhibiting stronger inhibition [25].

Despite the numerous benefits of silica fume and fly ash in autoclaved concrete products, GBFS remains the most widely used SCM in their production [26]. However, the supply of GBFS, particularly high-quality GBFS, is often limited relative to the enormous consumption of cement. The growing scarcity of high-quality GBFS has driven research into alternative industrial byproducts, including steel slag (SS) generated during steelmaking [6], ferronickel slag (FNS) produced in ferronickel alloy manufacturing [27], and lithium slag (LS) generated during lithium extraction processes [28]. However, the performance of these emerging SCMs relative to GBFS remains unclear. Specifically, their influence on cement hydration products and mechanical performance under autoclave curing conditions has not yet been fully elucidated.

Therefore, this study systematically investigates four SCMs (GBFS, SS, FNS, and LS) at a fixed 30% cement replacement level, with two core objectives: to characterize their influence on early-age hydration products under autoclave curing conditions and to quantify their impact on the development of mechanical properties. The findings will provide critical guidance for the utilization of industrial byproducts in autoclaved construction materials, addressing both performance requirements and sustainable material sourcing challenges.

## 2. Results and Discussion

### 2.1. Results of Material Characterization

#### 2.1.1. Chemical Composition Analysis

The chemical compositions of PC, FNS, LS, SS, and GBFS, determined through X-ray fluorescence (XRF) analysis, are presented in Table 1. FNS and SS contained relatively high levels of Fe_2_O_3_ and MgO. In contrast, LS was characterized by elevated concentrations of SiO_2_, Al_2_O_3_, and SO_3_, with the combined proportion of these three oxides reaching 91.23%. GBFS exhibited high levels of Al_2_O_3_ and SO_3_. Compared to SS and GBFS, FNS and LS had lower CaO contents. The Ca/Si molar ratio in the raw materials decreased in the order of PC > SS > GBFS > FNS > LS. Similarly, the Al/Si molar ratio followed the trend: GBFS > SS > LS > PC > FNS. Notably, LS contained the highest Al_2_O_3_ content among all materials, while GBFS ranked second. Moreover, in comparison to other SCMs, LS showed elevated concentrations of Na_2_O and K_2_O.

#### 2.1.2. Mineral Composition

**Figure 1:**
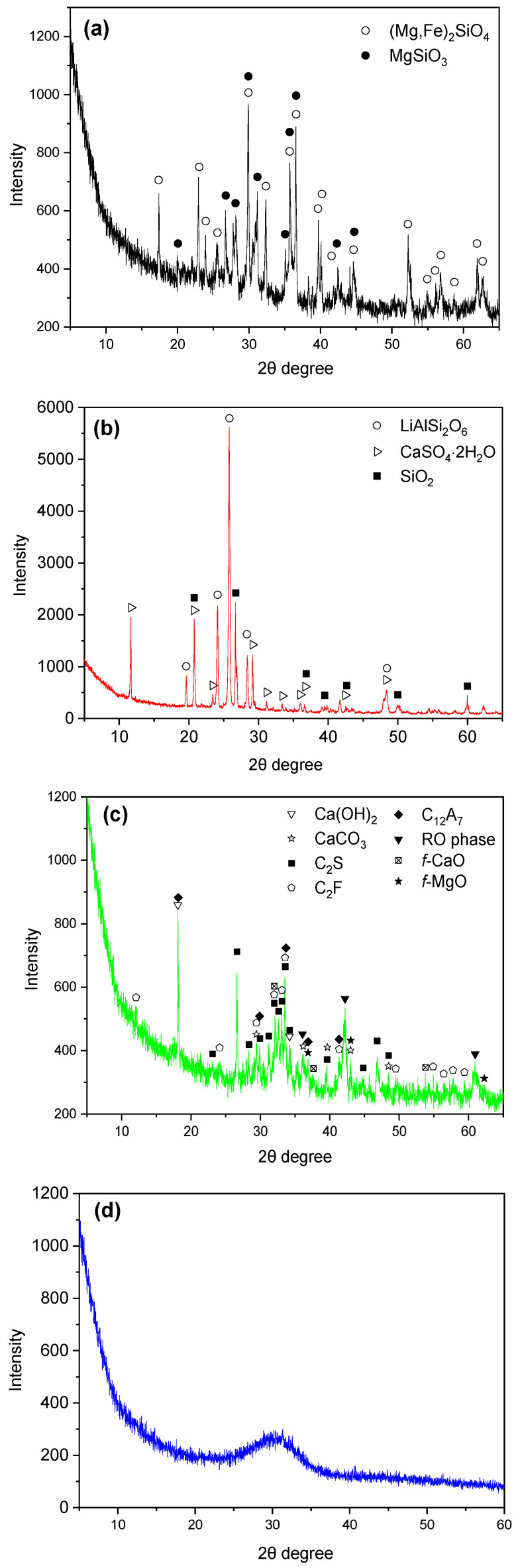
X-Ray diffraction pattern of SCMs: (**a**) FNS; (**b**) LS; (**c**) SS; (**d**) GBFS.

Figure 1 presents the XRD patterns of the mineral compositions of FNS, LS, SS and GBFS, with the results summarized in Table 2. With the exception of GBFS, the other three SCMs primarily consisted of crystalline phases, with relatively low amorphous content. In FNS, the dominant crystalline phases were forsterite ((Mg, Fe)_2_SiO_4_) and enstatite (MgSiO_3_), both of which exhibited high stability and minimal reactivity [27].

LS was mainly composed of spodumene (LiAlSi_2_O_6_), gypsum (CaSO_4_·2H_2_O), quartz and carbonates [28]. Although spodumene shows limited reactivity at ambient temperatures, its reactivity increases under elevated temperatures [28]. Gypsum is commonly used as a retarder in PC, whereas carbonates act as accelerators in cementitious systems [28].

The mineral composition of SS primarily consisted of C_2_S, C_2_F, C_12_A_7_, the RO phase, Ca(OH)_2_, CaCO_3_, *f*-CaO, and *f*-MgO. Among them, Ca(OH)_2_ and CaCO_3_ detected in SS are secondary products formed during its digestion and aging processes [29]. Despite this, free calcium oxide (*f*-CaO) and free magnesium oxide (*f*-MgO) are still detected in the steel slag, which can affect its volume stability. Therefore, special attention must be paid to its soundness in practical applications. Although SS contains C_2_S, a mineral phase also present in PC, its reactivity is significantly lower than that of PC [30].

#### 2.1.3. Particle Size Distribution

**Figure 2:**
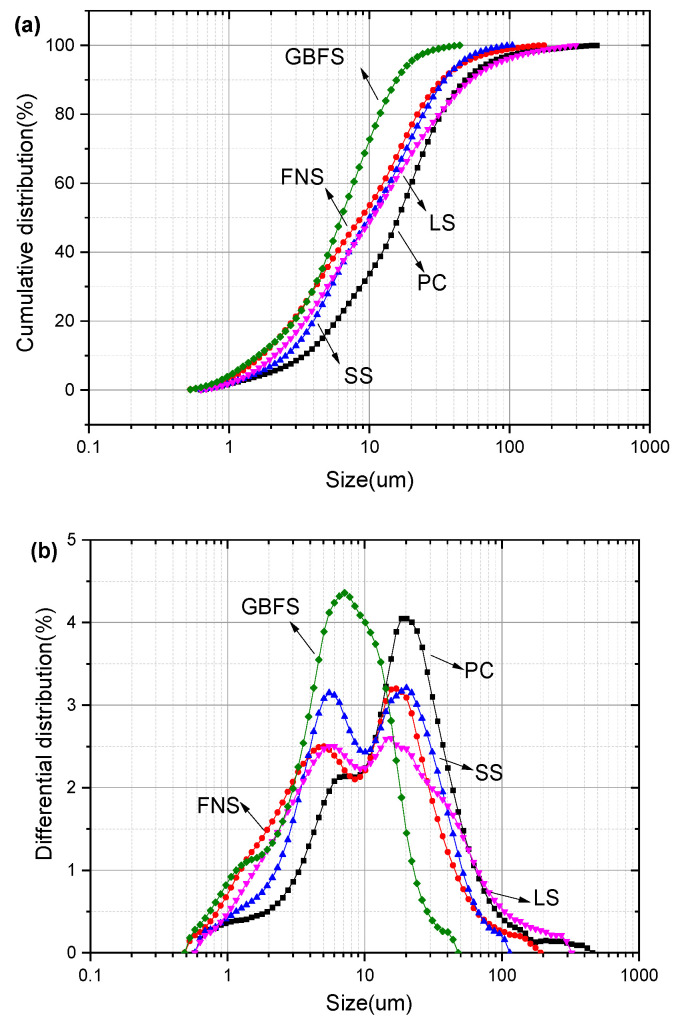
Particle size distribution of SCMs: (**a**) cumulative distribution (**b**) differential distribution.

The particle size distributions of SCMs as determined by laser particle size analysis, are presented in Figure 2. The d_10_ values of GBFS, FNS, SS, LS, and PC were 1.62, 1.76, 2.56, 2.11, and 3.27 μm, respectively; the d_50_ values were 6.36, 8.73, 10.03, 10.56, and 16.14 μm, respectively; and the d_90_ values were 15.63, 34.54, 34.21, 54.40, and 48.97 μm, respectively. Among these materials, PC exhibited the widest particle size distribution, while GBFS had the narrowest. In contrast, FNS, SS, and LS displayed similar particle size distributions, with each featuring two distinct peaks in their distribution curves.

#### 2.1.4. Thermal and Physical Characteristics

As presented in Table 2, SS demonstrated the highest loss on ignition (LOI) at 6.2%, primarily attributed to its Ca(OH)_2_ and CaCO_3_ content. LS followed with an LOI of 5.7%, resulting from its carbonate and gypsum composition. In comparison, PC, GBFS, and FNS exhibit significantly lower LOI values.

Regarding material densities, FNS and SS have higher values than GBFS and LS, which correlates with their elevated Fe_2_O_3_ and MgO contents. Among all materials, LS has the lowest density (2.60 g/cm^3^), marginally lower than that of GBFS.

#### 2.1.5. Morphological Analysis

**Figure 3:**
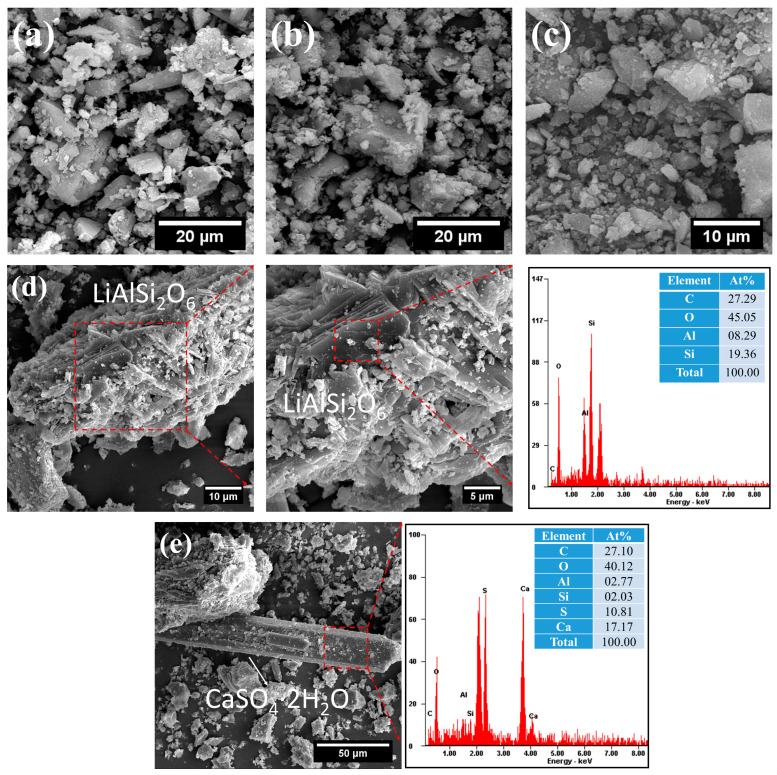
The morphology of SCMs under SEM: (**a**) FNS; (**b**) SS; (**c**) GBFS; (**d**) layered spodumene in LS; (**e**) rod-like gypsum in LS. Note: The elements are listed in the table in the order of their characteristic peaks appearing in the EDS spectrum, not in order of weight or atomic percentage.

As illustrated in Figure 3, FNS, SS, and GBFS predominantly consisted of irregular, gravel-like particles. Notably, LS displayed a distinct lamellar and rod-like morphology, attributable to its characteristic layered spodumene and elongated gypsum crystal structures [28]. It should be noted that lithium (Li) is the lightest metallic element, and its presence cannot be detected by EDS testing. Therefore, no characteristic signal of Li is observed in the EDS results of spodumene (Figure 3d).

#### 2.1.6. Reactivity Considerations

Notably, unlike SS and GBFS, neither FNS nor LS exhibits inherent hydraulic activity, which means they cannot react with water alone.

### 2.2. Water Demand, Setting Time, Soundness and Fluidity

The influence of a 30% cement replacement with FNS, LS, SS, and GBFS on properties including water demand, setting time, soundness, and mortar fluidity is summarized in Table 3. In Table 3, the notations Ref, F30, L30, S30, and G30 correspond to the plain cement reference and the blended cement mixtures containing 30% of FNS, LS, SS, and GBFS, respectively. The results demonstrated that both LS and GBFS significantly increased the water demand of cement paste, which can be primarily explained by their high specific surface areas. LS exhibited particularly high-water demand owing to two key factors. First, it has a porous microstructure resulting from the presence of layered spodumene and gypsum; second, its sulfate and carbonate components react rapidly with aluminates from cement to form ettringite, which consumes substantial amounts of free water [28]. In contrast, FNS and SS showed minimal effects on mortar fluidity.

Despite its high MgO content, the soundness test results revealed that FNS had minimal detrimental effects on cement stability. This observation stems from the fact that the MgO in FNS predominantly exists in stable crystalline forms (forsterite and enstatite) [27] or amorphous phases [31], rather than as *f*-MgO. Similar to observations in GBFS systems, the amorphous MgO present in FNS shows no measurable impact on soundness properties of concrete. However, SS-containing cement showed more noticeable soundness issues due to the presence of *f*-CaO, though the measured values remain within the acceptable limits specified by GB/T 1346-2011 [32].

Setting time measurements indicated significant differences among the various SCMs. FNS demonstrated a strong retarding effect owing to its low reactivity, while GBFS, with its higher pozzolanic activity, showed less influence on setting behavior. SS-modified cement exhibited the longest setting times, which can be attributed to the low reactivity of its mineral phases, even though SS has a large specific surface area [33,34,35]. LS displayed the least change in setting time compared to plain cement, which is a consequence of the counterbalancing effects between its gypsum content (retarding) and carbonate components (accelerating) [28].

### 2.3. XRD Analysis of Hydration Products

#### 2.3.1. Hydration Products Under Standard Curing Condition

**Figure 4:**
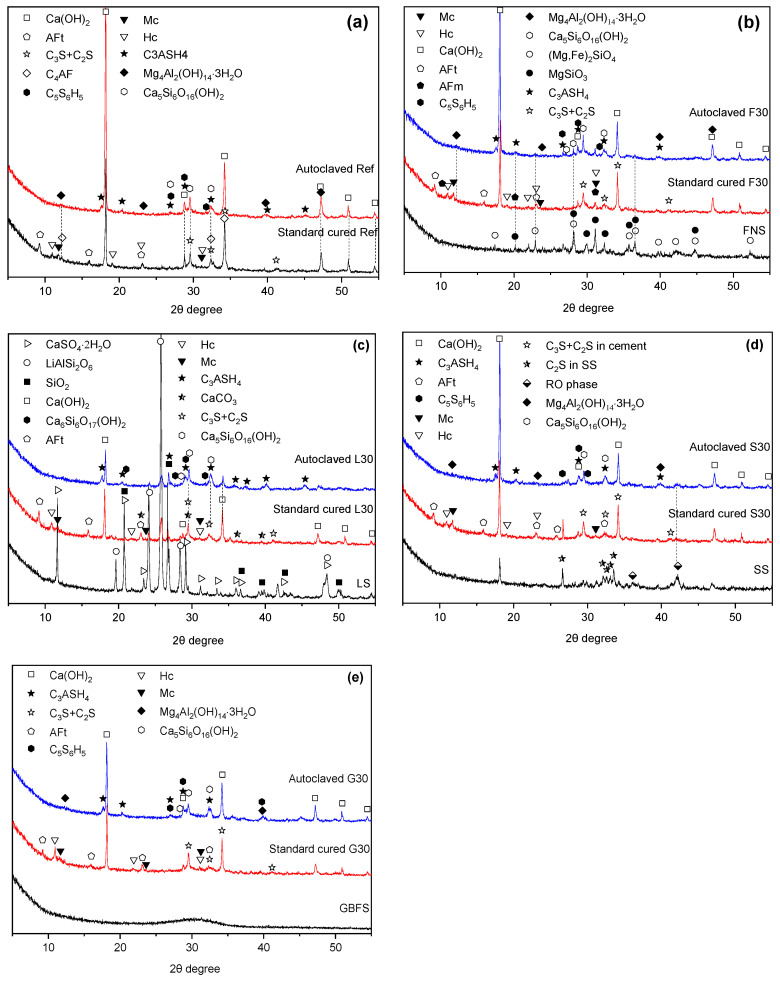
XRD of hydration products in (**a**) Ref, (**b**) F30, (**c**) L30, (**d**) S30 and (**e**) G30.

Figure 4 presents the XRD patterns comparing the hydration products in cement pastes with and without SCMs under both autoclave and standard curing conditions. After 28 days of standard curing, in pure cement, the primary crystalline hydration products were calcium hydroxide (CH), ettringite (AFt), monocarbonate (Mc), and hemicarbonate (Hc).

However, the incorporation of SCMs, did not significantly alter the types of hydration products formed. The low SO_3_ content in FNS promoted the formation of AFm in F30. In contrast, the elevated SO_3_ and carbonate levels in LS enhanced ettringite formation in L30. Furthermore, due to the presence of CaCO_3_ in SS [36], S30 favored Mc formation, whereas G30 tended to facilitate Hc formation.

#### 2.3.2. Hydration Products Under Autoclave Curing

(1) Comparison of hydration products: autoclaved vs. standard curing

Significant differences were observed in the hydration products formed under autoclave curing compared to standard curing conditions. XRD analysis revealed that autoclaving at 180 °C promoted the formation of crystalline C−S−H phases (e.g., tobermorite and riversideite), hydrogarnet and hydrotalcite. It also inhibited the formation of AFt, AFm phases, and monocarbonate/hemicarbonate hydrates due to their thermal instability at elevated temperatures [37].

Notably, the CH content exhibited distinct trends depending on the binder system. In pure cement and F30/S30/G30 blended systems, autoclave curing significantly enhanced CH formation, as evidenced by stronger diffraction peaks. Conversely, the L30 system showed reduced CH content, attributable to the accelerated pozzolanic reaction between LiAlSi_2_O_6_ and CH under autoclaved conditions [28]. This is further confirmed by the diminished LiAlSi_2_O_6_ diffraction peaks in autoclaved L30 samples, indicating improved reactivity of this phase at high temperatures.

(2) The impact of FNS

XRD analysis demonstrated that the crystalline hydration products formed in FNS-blended cement under autoclave conditions were compositionally analogous to those in pure cement systems. Both systems formed characteristic phases including tobermorite (Ca_5_Si_6_O_17_·5H_2_O), riversideite (Ca_5_Si_6_O_16_(OH)_2_), hydrogarnet (C_3_ASH_4_), hydrotalcite (Mg_4_Al_2_(OH)_14_·3H_2_O), and CH, which confirms that FNS incorporation did not significantly alter the phase composition under autoclave conditions.

However, comparative analysis revealed a distinct difference between autoclaved and standard-cured FNS-cement systems. The exclusive formation of hydrotalcite in autoclaved specimens suggests autoclave conditions enhanced the activation of MgO from both the cement matrix and the amorphous fraction of FNS. In contrast, the crystalline forsterite (Mg_2_SiO_4_) and enstatite (MgSiO_3_) phases demonstrated remarkable chemical inertness, which can be attributed to their thermodynamically stable structures and high activation energy barriers [27].

Hydrotalcite, a layered double hydroxide (LDH) compound, exhibits unique physicochemical properties that make it particularly suitable for concrete applications. Its characteristic layered structure and high specific surface area give it exceptional ion-exchange capacity and selective adsorption capabilities. This enables effective immobilization of aggressive species including chloride (Cl^−^) and sulfate (SO_4_^2−^) [38,39]; therefore, the formation of hydrotalcite contributes to enhancing the durability of concrete.

(3) The impact of LS

Phase evolution analysis revealed distinct differences between autoclaved L30 and the reference sample. Although tobermorite formation was limited in L30, xonotlite (Ca_6_Si_6_O_17_(OH)_2_) became the predominant crystalline phase. This observation aligns with previous studies demonstrating that elevated temperatures (>150 °C) promote the transformation of tobermorite to xonotlite [19]. Additionally, Taylor found that at a curing temperature of 60 °C, the primary hydration product of cement is 14 Å tobermorite, whereas at 100 °C, the main hydration product is 11 Å tobermorite [40,41,42]. Based on these findings, he hypothesized that higher temperatures favor the formation of hydration products with lower water content [40,41,42]. As depicted in Figure 4, the high aluminum content in LS makes the formation of hydrogarnet highly favorable under autoclave curing conditions. This formation process consumes a substantial amount of water. As a consequence, conditions are created that facilitate the transformation of crystalline C−S−H from high water content phases (tobermorite) to low water content phases (xonotlite).

Additionally, the dilution effect of LS (30% replacement) reduced the system’s MgO availability, suppressing hydrotalcite formation.

(4) The impact of SS and GBFS

Under autoclave curing conditions, the hydration products of S30 and G30 exhibited substantial similarity to those of the reference sample (Ref). It is noteworthy that a portion of MgO, derived from both *f*-MgO and the RO phase in SS, participated in the hydration process, leading to the formation of hydrotalcite. This is consistent with the conclusion of literature [6]. This phenomenon can be attributed to the enhanced reactivity of MgO-containing phases under the elevated temperature and pressure conditions.

### 2.4. TG/DTG Analysis of Hydration Products

#### 2.4.1. Hydration Products Under Standard Curing Condition

**Figure 5:**
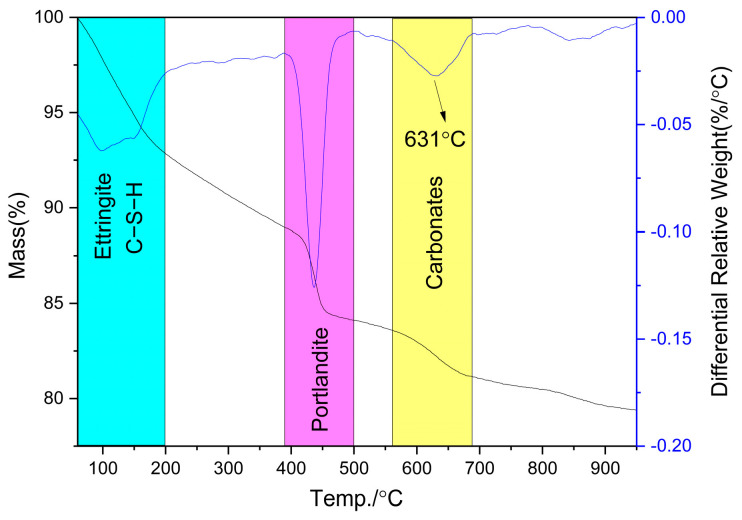
TG/DTG of hydration products in Ref under standard curing condition.

Figure 5 shows the TG and DTG curves of Ref cured under standard conditions for 28 days. Since SCMs have little effect on the types of cement hydration products, and existing literature [30,31,37,43] has already reported TG/DTG curves for blends containing FNS, LS, SS, and GBFS, this study did not provide additional data on these admixtures.

The TG-DTG analyses indicated that the main hydration products of Ref after 28 days of standard curing included C−S−H, CH, ettringite, and a small amount of calcite. The formation of calcite was relatively limited and was not clearly detected in the XRD pattern. Therefore, its characteristic peaks are absent in the XRD results shown in Figure 4.

In addition, research indicated that the mass loss in the 60~600 °C range typically reflects the non-evaporable water content in cement paste [44]. Figure 5 and literature data [30,31,37,43] show that the non-evaporable water in composite cements containing 20%FNS, LS, SS, and GBFS was 85.6%, 91.9%, 95.0%, and 89.6% of that Ref, respectively. Meanwhile, their CH contents were 83.5%, 84.0%, 94.6%, and 66.5%, respectively. Interestingly, the strength activity index (defined as the ratio of the 28-day strength of specimens containing SCMs to that of the plain cement specimens at an equal replacement level) ranked as GBFS > LS > SS > FNS under standard curing conditions. However, this order showed no correlation with their non-evaporable water content. This discrepancy may be attributed to varying degrees of pozzolanic reaction between the SCMs and CH: highly reactive SCMs consume more CH, potentially leading to reduced non-evaporable water.

Further analysis revealed that the “effective non-evaporable water” (60~390 °C range), obtained by excluding the contribution of CH, exhibited a stronger correlation with mechanical performance of mortar with SCMs, particularly at higher SCMs dosages. After recalculation, the effective non-evaporable water in composite cements with 20% FNS, LS, SS, and GBFS was 87.8%, 97.4%, 95.2%, and 98.9% of that in Ref, respectively. This parameter more accurately reflected the mechanical properties of mortar.

#### 2.4.2. Hydration Products Under Autoclave Curing Condition

(1) Types of hydration products

**Figure 6:**
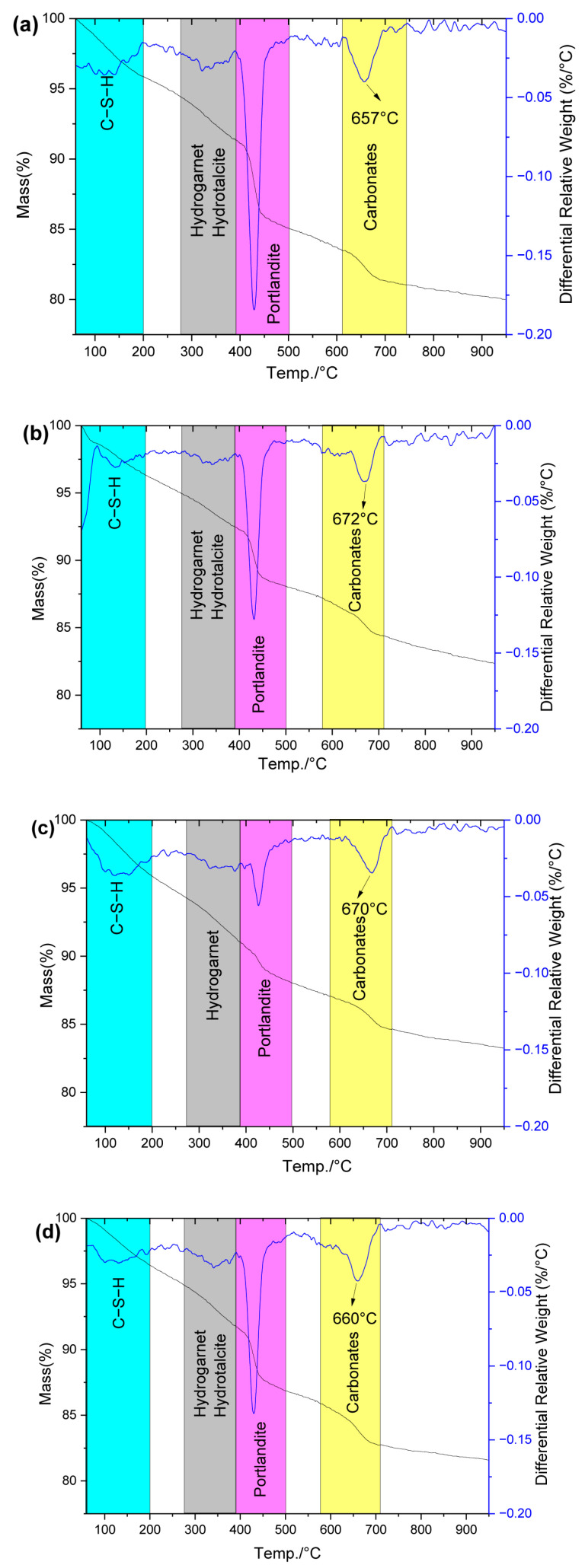

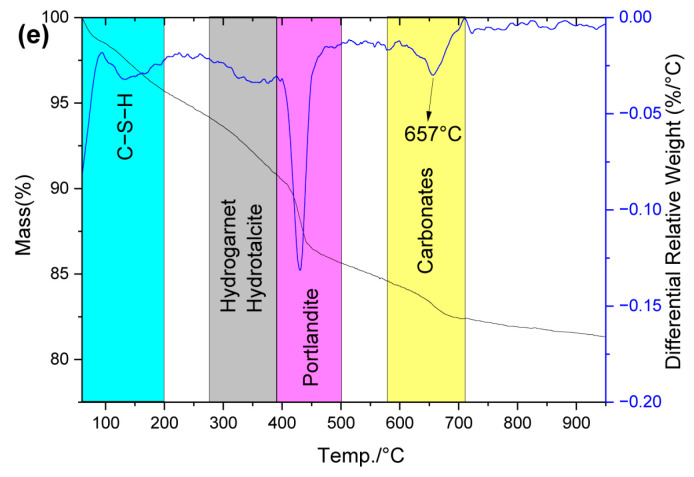
TG/DTG of hydration products in (**a**) Ref, (**b**) F30, (**c**) L30, (**d**) S30 and (**e**) G30 under autoclave curing condition.

To further assess the effect of SCMs on hydration product formation under autoclave curing, TG/DTG analyses were conducted on all hardened cement pastes (Figure 6). Autoclave-cured cement pastes exhibited similar hydration products to standard curing, except for hydrogarnet and hydrotalcite formation. Notably, although FNS contains amorphous MgO and Fe_2_O_3_, its incorporation did not change the hydration product types because: (1) Cement-derived MgO already participates in hydration product formation under autoclave curing conditions; (2) Amorphous Fe_2_O_3_ in FNS reacts slowly, behaves similarly to Al_2_O_3_, and can replace Al_2_O_3_ to participate in calcium sulfoaluminate/aluminate hydrate formation. It is reported that the reactivity of the Fe_2_O_3_-containing phase in cementitious systems depends on the Al_2_O_3_/Fe_2_O_3_ molar ratio [45]. In FNS, this ratio is relatively low due to its minimal Al_2_O_3_ content, which is comparable to that of PC. As a result, the reactivity of Al_2_O_3_ in FNS remains limited.

Similarly, LS, SS, and GBFS incorporation maintained identical hydration product types under autoclave curing.

(2) Content of C−S−H and hydrogarnet

Using the mass loss data obtained from Figure 5 and Figure 6, the total mass loss and the differential mass losses in key temperature ranges (<200 °C, 280~390 °C, <390 °C, 390~500 °C, and 600~800 °C) were calculated. Notably, the mass losses in the 390~500 °C and 600~800 °C ranges are specifically attributed to CH and CaCO_3_ decomposition [46], respectively, as summarized in Table 4. The lower temperature ranges provided additional information about hydrates: mass loss below 200 °C mainly corresponds to the bound water from C−S−H, while the 280~390 °C range mainly represents hydrotalcite and hydrogarnet phases [37,47,48].

The analysis of Table 4 indicated that at temperatures below 390 °C, the effective non-evaporable water content in G30, L30, S30, and F30 corresponded to 106.5%, 104.9%, 95.7%, and 87.2% of Ref, respectively. The observation reflects the progressively decreasing reactivity of SCMs (GBFS > LS > SS > FNS) under autoclave curing conditions. In the <200 °C range (C−S−H content), the mass loss decreased in the following order: G30 (104.6%), L30 (100.2%), F30 (89.6%), and S30 (87.0%) relative to Ref. Notably, F30 surpassed S30 in this range, which can be attributed to the enhanced formation of hydrogarnet and hydrotalcite (observed in the 280~390 °C range) in S30 under autoclave curing conditions.

The observed variations in hydrotalcite and hydrogarnet formation can be explained by the differing reactive Al_2_O_3_ and MgO contents in each SCM. Specifically, GBFS-containing samples (G30) showed the highest phase content due to their elevated Al_2_O_3_ and MgO levels (Table 1), whereas FNS samples (F30) exhibited minimal formation. Interestingly, despite its intermediate position in the reactivity series, the lithium slag sample (L30) demonstrated substantial hydrogarnet formation, attributable to its characteristically high Al_2_O_3_ content.

(3) CH Content

Under autoclave curing conditions, significant changes occurred in the CH content of cement paste. Compared to standard 28-day curing, autoclaved cement produced fewer hydration products overall, yet interestingly more CH was generated in Ref, F30, S30, and G30, as shown in Table 4 and Figure 4. This indicates that, compared to standard-cured samples, the autoclave-cured samples actually exhibit a lower hydration degree. However, it should also be noted that a higher CH content is more susceptible to chemical attack by corrosive ions, such as sulfate ions [49].

The autoclave-cured cement pastes exhibited decreasing CH content in the order: Ref > G30 > S30 > F30 > L30. Among these, the CH contents in G30, S30, F30, and L30 were only 82.4%, 77.9%, 70.1%, and 47.7% of that in Ref, respectively. The occurrence of this phenomenon is attributed to the fact that most SCM components become reactive under autoclave curing conditions. In these composite cements, C−S−H formation derives CaO from both cement and SCMs. When the SCM replacement levels are equal, those with higher CaO content (GBFS and SS, as shown in Table 1) require less CaO from cement to form equivalent C−S−H, leaving more available for CH formation. Consequently, G30 (containing CaO-rich GBFS) shows the highest CH content, while L30 (with CaO-poor LS) displays the lowest.

Notably, S30 demonstrated slightly lower CH content than G30 despite SS’s high CaO content. This apparent anomaly likely results from pronounced carbonation in the steel slag-blended paste, which converts more CH into CaCO_3_ (Table 4).

### 2.5. SEM-EDS Analysis of Hydration Products

#### 2.5.1. Hydration Products of Cement Paste After 28d Standard Curing

(1) C−S−H

**Figure 7:**
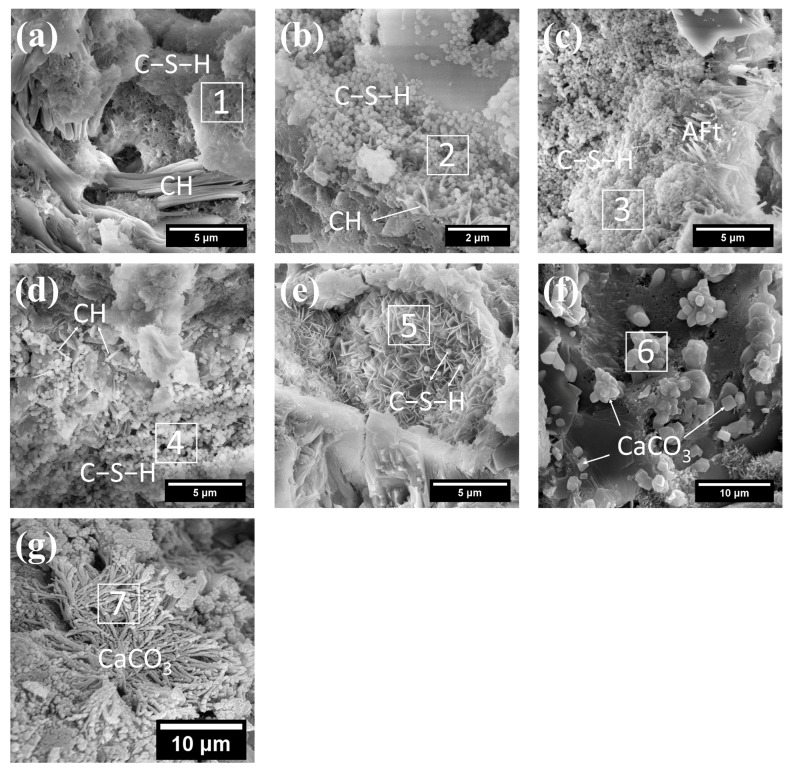
Morphology of hydration products of standard cured cement paste: (**a**) C−S−H and CH in Ref; (**b**) C−S−H and CH in F30; (**c**) C−S−H and AFt in L30; (**d**) C−S−H and CH in S30; (**e**) C−S−H in G30; (**f**) CaCO_3_ in L30; (**g**) CaCO_3_ in G30.

To explore the effects of SCM on the hydration products of autoclave-cured concrete, this study first investigated their influences on the morphology and composition of cement hydration products under standard curing conditions for 28 days. The corresponding results are illustrated in Figure 7, with the compositional details of hydration products summarized in Table 5. As presented in Figure 7 and Table 5, the microstructural and compositional analyses revealed significant modifications induced by SCMs incorporation. The reference sample (pure cement) displayed characteristic reticular C−S−H gels with a Ca/Si ratio of 2.71 and minimal trace elements (Figure 7a). However, upon SCM addition, the hydration products displayed a bimodal morphology, combining the original reticular structure with newly formed spherical particles (Figure 7b,d). This morphological transformation clearly demonstrated SCMs’ substantial influence on hydration mechanisms. The spherical C−S−H particles, resulting from pozzolanic reactions between SCMs and cement-derived CH, exhibited significantly lower Ca/Si ratios (0.52 for F30, 1.65 for L30, and 1.44 for S30). Moreover, elemental analysis revealed enhanced aluminum incorporation in SCM-modified samples, with Al/Si ratios of 0.14 (Ref), 0.39 (L30), 0.28 (S30), and 0.22 (G30).

As the main hydration product of cement, the C−S−H gel does not have a single, fixed structure. Instead, it is highly amorphous and exhibits diverse morphologies across different scales [50,51]. Generally, C−S−H presents reticular, honeycomb-like, fibrous, porous, and flake-like morphologies [52]. The formation of such morphological features is mainly influenced by several key factors: hydration age, water-to-cement ratio, type of SCMs, hydration environment, and chemical additives. These spherical substances only appear in blended cement with SCMs, but not in plain cement. Furthermore, they are only formed at a specific curing age (e.g., 28 days) and are not detected in the early and longer curing stages [30,31,37,43]. These evidence confirms that these spherical substances are a type of metastable C−S−H gel formed at a specific curing age after the hardening of cement paste with SCMs added.

It is worth noting that, compared to traditional amorphous C−S−H gels, the spherical C−S−H particles exhibited a larger specific surface area and a more uniform distribution morphology. These newly formed spherical particles filled the pores within fibrous C−S−H, thereby increasing the compactness of C−S−H and showing potential to improve the long-term mechanical properties and durability of concrete. Research indicates that the fundamental structural unit of C−S−H is nanoscale spherical particles (approximately 4.4 nm in size) [53,54,55]. Essentially, C−S−H is a porous gel system formed by the fractal packing of such spherical units, and its mechanical performance is primarily governed by the packing density of the spherical particles [53,54,55]. In this study, the observed spherical C−S−H particles were larger, ranging from approximately 100 to 200 nm in size. They were likely formed through agglomeration of the fundamental structural units and could be regarded as an intermediate transitional form between the basic structural units of C−S−H gels and traditional fibrous C−S−H or lamellar C−S−H gels.

Microstructural analysis of G30 (Figure 7e) revealed not only spherical C−S−H but also flaky C−S−H formations, both exhibiting similarly low Ca/Si ratios. This observation, combined with the known tendency of GBFS to promote hydrotalcite formation [37], suggested complex hydration pathways in GBFS-blended systems.

(2) CH

Furthermore, the characteristic CH crystals in pure cement paste (5–20 μm, Figure 7a) underwent dramatic size reduction (1–2 μm) upon SCM incorporation (Figure 7b,d), accompanied by decreased crystal quantities. These concurrent changes demonstrated SCMs’ dual influence on CH formation.

SCMs can reduce the content of CH in hardened cement paste, primarily due to the combined effects of the pozzolanic reaction and dilution effect [56,57]. While, the refining effect of SCMs on CH crystal size is mainly accomplished through two mechanisms: (1) Fine SCM particles provide abundant heterogeneous nucleation sites for CH crystallization. These additional sites facilitate the simultaneous crystallization of CH at multiple locations, instead of directional growth at only a few sites, thus effectively suppressing the excessive growth of individual CH crystals. (2) Fine SCM particles act as physical barriers to directly limit the growth space of CH crystals. Furthermore, the filling effect of SCM particles optimizes the particle packing structure of the paste, reduces capillary pore size, and imposes a secondary restriction on the growth space of CH crystals.

(3) CaCO_3_

Microstructural characterization of the L30 sample (Figure 7c) demonstrated two distinct features: first, the predominant formation of rod-like ettringite crystals, which directly correlated with the elevated sulfate content derived from gypsum in LS; second, a significant increase in the content of cubic CaCO_3_ phases (Figure 7f). The latter observation can be attributed to both the intrinsic carbonate content of LS and partial carbonation that occurred during sample handling and preparation [28].

Notably, CaCO_3_ in G30 developed a distinct fibrous morphology (Figure 7g), contrasting sharply with the cubic forms observed in L30. This morphological divergence demonstrated the high sensitivity of CaCO_3_ crystallization pathways to local chemical environments, specifically the ionic composition and alkalinity (pH 10–12) of the pore solution during precipitation [37,58].

CaCO_3_ commonly exists in three crystalline polymorphs: calcite, aragonite, and vaterite. Among them, calcite is the most stable crystalline form, with typical rhombohedral or cubic morphologies [59]. Aragonite is commonly observed with needle-like, columnar or hexagonal prismatic morphologies. In contrast, as the least stable polymorph, vaterite generally appears as spherical particles or irregular aggregates [60,61]. It typically forms in the early hydration stage or under specific conditions and subsequently transforms into the more stable calcite or aragonite.

Factors influencing the crystalline form and morphology of CaCO_3_ include supersaturation, pH value, temperature, reaction time, stirring rate, feeding sequence of reaction solutions, and the type and dosage of additives [37,58]. The low alkalinity and high impurity content of lithium slag are probably the main reasons for the difference in the morphology of CaCO_3_ between the lithium slag composite cement system and GBFS composite cement system.

#### 2.5.2. Hydration Products of Cement Paste Under Autoclave Curing Conditions

(1) C−S−H

**Figure 8:**
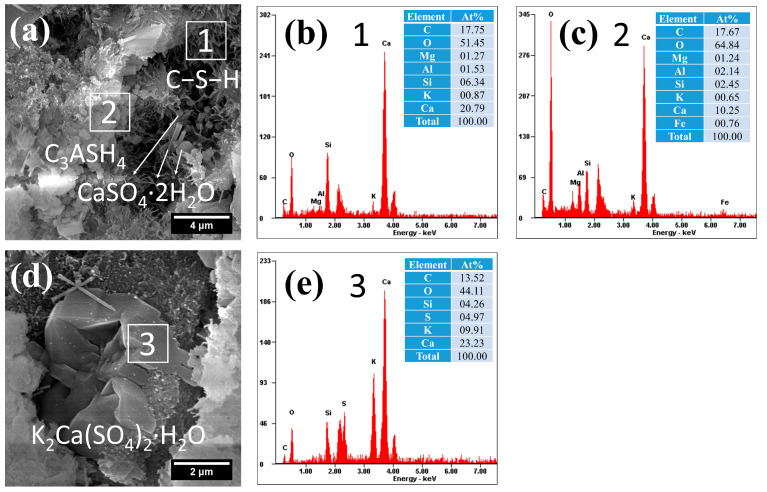
Morphology and composition of hydration products in autoclaved Ref: (**a**) C−S−H and hydrogarnet; (**b**) composition of C−S−H in area 1; (**c**) composition of C_3_ASH_4_ in area 2; (**d**) potassium gypsum; (**e**) composition of potassium gypsum in area 3.

**Figure 9:**
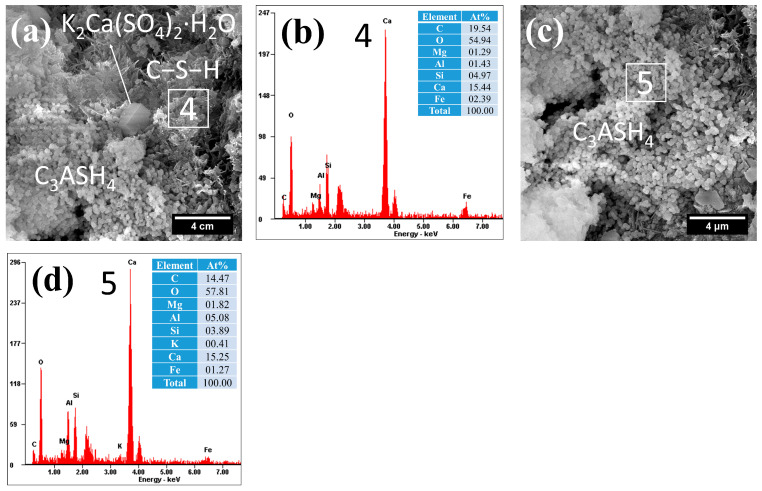
Morphology and composition of hydration products in autoclaved F30: (**a**) C−S−H and potassium gypsum; (**b**) composition of C−S−H in area 4; (**c**) Hydrogarnet; (**d**) composition of C_3_ASH_4_ in area 5.

**Figure 10:**
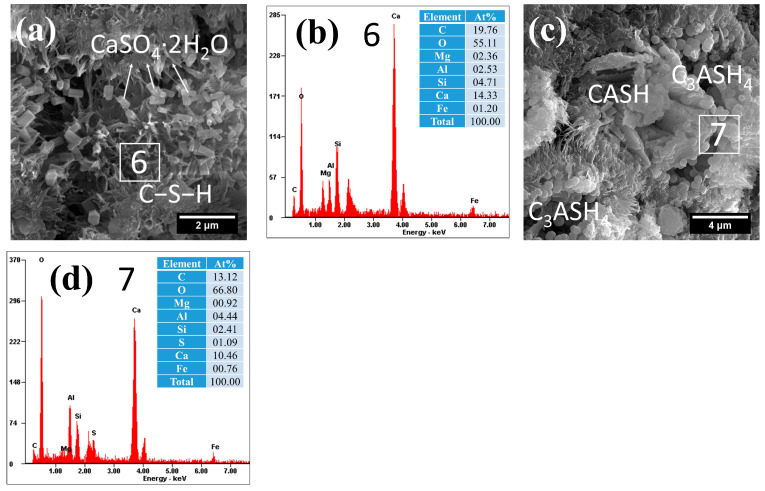
Morphology and composition of hydration products in autoclaved L30: (**a**) C−S−H and gypsum; (**b**) composition of C−S−H in area 6; (**c**) CASH and hydrogarnet; (**d**) composition of C_3_ASH_4_ in area 7.

**Figure 11:**
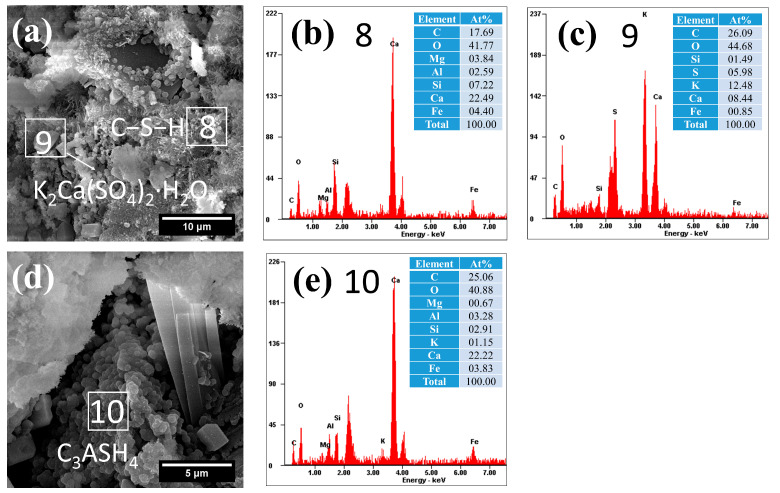
Morphology and composition of hydration products in autoclaved S30: (**a**) C−S−H and potassium gypsum; (**b**) composition of C−S−H in area 8; (**c**) composition of potassium gypsum in arae 9; (**d**) hydrogarnet; (**e**) composition of C_3_ASH_4_ in area 10.

**Figure 12:**
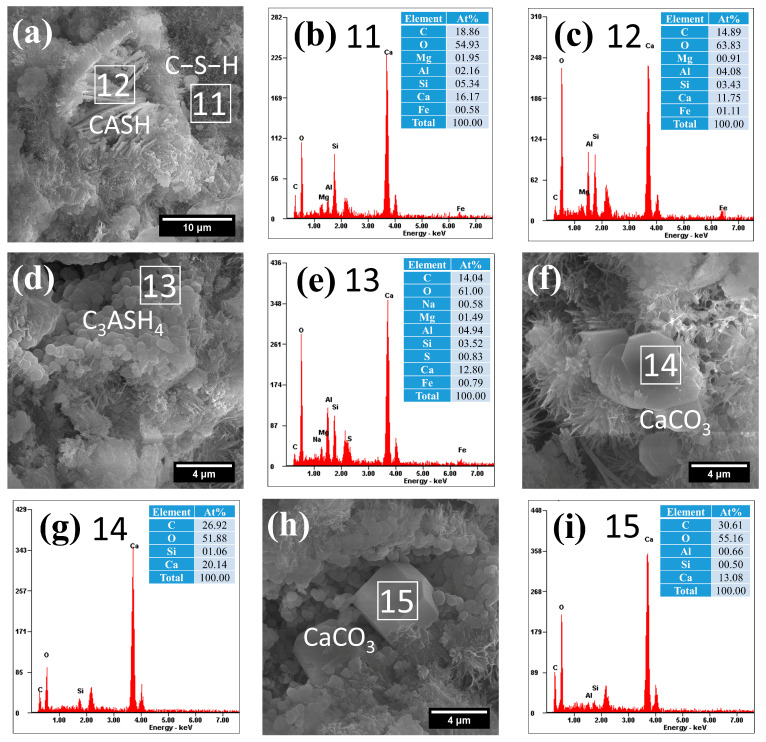
Morphology and composition of hydration products in autoclaved G30: (**a**) C−S−H and hydrogarnet; (**b**) composition of C−S−H in area 11; (**c**) composition of CASH in area 12; (**d**) hydrogarnet; (**e**) composition of C_3_ASH_4_ in area 13; (**f**) CaCO_3_; (**g**) composition of CaCO_3_ in area 14; (**h**) CaCO_3_; (**i**) composition of CaCO_3_ in area 15.

The microstructural and compositional characteristics of hydration products in autoclave-cured cement pastes are systematically presented in Figure 8, Figure 9, Figure 10, Figure 11 and Figure 12. Distinct from standard-cured specimens, autoclave conditions promoted the formation of fibrous C−S−H with reduced interconnectivity. Compared to traditional reticulated C−S−H, this fibrous C−S−H may weaken the bonding capacity of the cementitious matrix. This morphological change was accompanied by a characteristically elevated Ca/Si ratio in the C−S−H phase, which measured 3.28 in the reference sample (area 1 in Figure 8) and ranged between 3.03 and 3.11 in SCM-modified pastes (F30: area 4 in Figure 9a; L30: area 6 in Figure 10a; S30: area 8 in Figure 11a; G30: area 11 in Figure 12a). These high Ca/Si ratios collectively indicated a lower hydration degree of cement under autoclave conditions when compared to standard 28-day curing.

(2) Hydrogarnet

Under autoclave curing conditions, all cement pastes formed a significant amount of spherical hydration products, which exhibited a morphology similar to that of spherical particle-like C−S−H, as shown in Figure 8, Figure 9, Figure 10, Figure 11 and Figure 12. However, these hydration products exhibited significantly larger particle sizes (approximately 0.5 μm), and their dimensions increased with the Al_2_O_3_ content in the SCMs, for example, L30 (Figure 10c), S30 (Figure 10d) and G30 (Figure 12d) showed larger particle sizes compared to F30 (Figure 9c). This characteristic distinguished them from typical C−S−H. Additionally, their Al/Si ratios were notably higher, measuring 0.87, 1.31, 1.84, 1.13, and 1.40, respectively, which were closer to 1. These elevated Al/Si ratios, combined with their distinct spherical morphology, suggested a composition and structure that differed significantly from conventional C−S−H. Given these characteristics, including their morphology and Al/Si ratios, the spherical hydration products closely resembled those of hydrogarnet (C_3_ASH_4_). Therefore, it can be inferred that these spherical hydration products are indeed hydrogarnet.

The formation of the hydrogarnet phase predominantly occurs through the transformation of metastable calcium aluminate hydrate phases (CAH_10_, C_2_AH_8_, and C_4_AH_13_), as extensively documented in previous studies [62]. This transformation kinetics exhibits marked temperature sensitivity, and elevated curing temperatures dramatically promote the conversion rate. More notably, this phase transformation induces adverse microstructural modifications, specifically, a reduction in solid-phase volume coupled with concomitant pore formation [63]. In turn, these microstructural changes may ultimately lead to a significant decline in the long-term strength of autoclave-cured concrete.

(3) Gypsum-like products

Under autoclave curing conditions, prismatic crystalline phases were observed in the cement paste (Figure 8, Figure 9, Figure 10 and Figure 11). EDS analysis revealed high sulfur (S) and potassium (K) concentrations, indicating the possible formation of gypsum (CaSO_4_·2H_2_O) or syngenite (K_2_Ca(SO_4_)_2_·H_2_O), as evidenced in area 3 in Figure 8d, Figure 9a and Figure 10a and area 9 in Figure 11a.

Although the autoclaved curing temperature reaches 180 °C, at which CaSO_4_·2H_2_O can dehydrate to form CaSO_4_·0.5H_2_O [64]. However, the specimens undergo sequential cooling and ambient-temperature pretreatment steps (including specimen preparation, grinding, storage, etc.) prior to testing. During these processes, the CaSO_4_·0.5H_2_O generated by dehydration rehydrates with environmental moisture, regenerating CaSO_4_·2H_2_O. This phase formation is attributed to the curing regime, namely standard curing followed by autoclave curing. The high-temperature and high-pressure conditions during autoclave curing decompose the ettringite-like phases generated during initial hydration, thus releasing sulfate ions into the pore solution. These sulfate ions may either remain dissolved in the pore solution, adsorb onto the surfaces of C−S−H gels, or reprecipitate in the form of gypsum or syngenite [65].

Subsequent exposure to ambient wet curing may induce internal sulfate attack, where residual gypsum reacts with aluminate hydrates in the presence of Ca(OH)_2_, forming secondary ettringite [66]. This delayed ettringite formation (DEF) can cause expansive stresses, leading to microcracking and reduced durability [67].

Notably, L30 appeared to form more gypsum under autoclave curing conditions (Figure 10a), attributable to: (i) the inherent gypsum content in LS and (ii) substantial ettringite generation during prior standard curing. These findings are corroborated by compositional (Table 1) and microstructural (Figure 7c) analyses.

(4) Crystalline CASH

The elevated Al_2_O_3_ content in both LS and GBFS facilitated the formation of crystalline calcium aluminosilicate hydrate (CASH) under autoclave curing conditions, as demonstrated in the L30 and G30 samples (Figure 10c and Figure 12a). These phases exhibited typical lamellar or plate-like morphologies, which are consistent with the research findings in the literature [68]. The formation of the crystalline CASH phase may increase the porosity of hardened cement paste and affect its bonding with aggregates.

Notably, the CASH phases demonstrated significantly elevated Al/Si ratios under autoclave curing, with G30 reaching an Al/Si ratio of 1.19 (area 12 in Figure 12a). This is not only related to the higher Al_2_O_3_ content in the precursor material but also to the higher curing temperature and pressure.

(5) CaCO_3_

In addition, autoclave curing induced the formation of plate-like (area 14, Figure 12f) and polyhedral (area 15, Figure 12h) CaCO_3_ crystals in the composite cement pastes, exhibiting markedly different morphologies from those formed under standard curing (Figure 7f,g). This distinct crystallization behavior underscores the profound impact of autoclave curing conditions on CaCO_3_ polymorph formation.

EDS analysis revealed the incorporation of trace Si and Al into the autoclave-formed CaCO_3_ structure, which may account for the observed morphological variations through their influence on crystal growth mechanisms. Furthermore, DTG analysis (Figure 5 and Figure 6) demonstrated a significant increase in CaCO_3_ decomposition temperature from 631 °C (standard curing) to 657–672 °C (autoclave curing), confirming structural modifications in the autoclave-derived CaCO_3_.

### 2.6. Mechanical Property

#### 2.6.1. Compressive Strength

**Figure 13:**
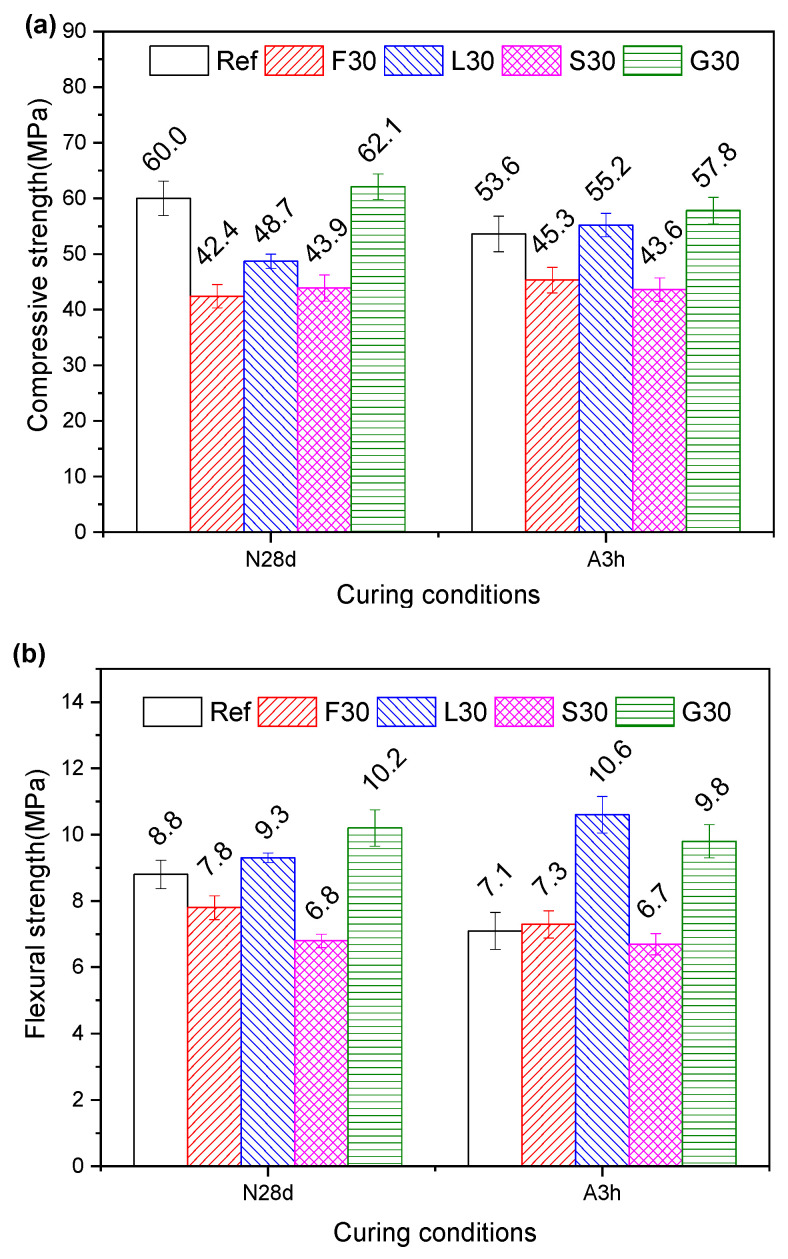
Strength of mortar samples: (**a**) compressive strength; (**b**) flexural strength, where N28d represents standard curing for 28 days, and A3h represents autoclave curing for 3 h.

Figure 13 compares the mechanical performance of cement mortars containing SCMs under both standard (28-day) and autoclave curing conditions. Among all mixtures, G30 achieved the highest compressive strength under both curing regimes, exceeding the reference sample (Ref). Conversely, F30 displayed the lowest strength under standard curing conditions, whereas S30 exhibited the poorest performance under autoclave curing.

Under standard curing, the compressive strength activity indices (defined as the ratio of the compressive strength of mortar containing SCMs to that of the reference mortar) were 70.7% (F30), 81.2% (L30), 73.2% (S30), and 103.5% (G30), revealing the following reactivity order: GBFS > LS > SS > FNS. Autoclave curing significantly altered this behavior, with indices increasing to 84.5% (F30), 103.0% (L30), 81.3% (S30), and 107.8% (G30). Notably, LS exhibited the most pronounced reactivity enhancement (21.8% increase), followed by FNS (13.8%), suggesting autoclave curing conditions preferentially activate these SCMs.

Although autoclave-cured specimens generally showed lower absolute strengths than their 28-day standard-cured counterparts (89.3~113.3% of reference values), two exceptions emerged: L30 and F30 demonstrated slightly higher strengths (106.8% and 113.3%, respectively) following autoclave treatment. This highlights how the activation potential of different SCMs depends on the curing method, particularly in LS and FNS systems.

It should be noted that although high-temperature curing accelerates the rapid formation of cement hydration products in a short time, these early-formed products tend to form a dense coating on the surface of unhydrated cement particles. This further hinders the contact between the particles and water, inhibits the progress of subsequent hydration reactions, and ultimately results in a reduction in strength [69,70].

#### 2.6.2. Flexural Strength

Under standard curing conditions, G30 demonstrated superior flexural strength with an activity index (defined as the ratio of the flexural strength of mortar containing SCMs to that of the reference mortar) of 115.9%, outperforming L30 (105.7%), F30 (88.6%), and S30 (77.3%). The consistently higher flexural strength activity indices compared to compressive strength values suggested that SCMs contribute to microstructural improvements beyond simple strength enhancement. This improvement mechanism involves dual effects: reduction in CH content and refinement of CH crystal size in the hardened paste [37], both of which contribute to enhanced interfacial transition zone (ITZ) quality and consequently improve flexural performance.

In contrast to standard curing, autoclave curing induced a significant shift in the flexural strength activity index of mortar. L30 exhibited the highest performance with an activity index of 149.3%, outperforming G30 (138.0%), F30 (102.8%), and S30 (94.4%). Although these indices generally exceeded their standard-cured counterparts, only L30 demonstrated absolute flexural strength surpassing its 28-day standard-cured strength value. This distinctive behavior likely stems from the thermal activation of spodumene and quartz phases within LS under autoclave curing conditions [28]. The high-temperature and high-pressure environment enhances the reactivity of these mineral components, resulting in substantial formation of C−S−H phases along with significant consumption of CH (Table 4), which partially offsets the detrimental effects typically associated with autoclave curing.

The observed strength behavior reveals a complex relationship between SCM composition and curing environment. Lithium slag’s exceptional performance under autoclave curing conditions, contrasted with its moderate performance under standard curing, highlights its particular suitability for high-temperature/high-pressure applications. These findings emphasize the critical importance of matching SCM selection with anticipated curing conditions to optimize mechanical performance in cementitious systems.

### 2.7. Discussion

#### 2.7.1. Hydration Products of Cement Paste Under Autoclave Curing Conditions

Under autoclave curing conditions, the increased crystallinity of C−S−H typically promotes the development of low-Ca/Si ratio phases (e.g., fibrous tobermorite). However, in our experimental system with mild autoclaving parameters (0.8 MPa, 3.0 h), XRD analysis (Figure 4) revealed only limited tobermorite formation. Instead, the dominant hydration products were conventional fibrous C−S−H gels (Figure 8, Figure 9, Figure 10, Figure 11 and Figure 12) exhibiting unusually high Ca/Si ratios (3.03–3.28)—significantly greater than those characteristic of tobermorite (typically 0.8–1.1) [11]. This deviation from expected phase composition strongly suggests that the reduced pressure and abbreviated curing duration inhibited the typical crystallization pathway, favoring the persistence of high-Ca/Si C−S−H structures.

Under conventional hydration conditions, the dissolution rate of calcium ions from cement clinker phases markedly exceeds that of silicate ions during the early hydration period [71,72,73]. As a result, the C−S−H gels formed in this calcium-rich environment displays an elevated Ca/Si ratio. Following initial standard curing and subsequent autoclave treatment, the difference in dissolution rates between calcium and silicate ions may be further amplified, causing the Ca/Si ratio in C−S−H to rise even higher. The C−S−H structure formed at this stage exhibits relatively low stability and consists predominantly of alternating C−S−H and CH layers. As hydration continues, the gradual dissolution of CH and the release of C−S−H layers lead to a progressive reduction in the Ca/Si ratio [71,72,73].

Interestingly, XRD analysis revealed complete absence of tobermorite in the autoclaved LS cement system, with xonotlite emerging as the predominant crystalline phase. This phase selection can be attributed to the system’s unique chemistry: (1) The elevated aluminum content in LS promotes extensive hydrogarnet formation in L30 mixtures, consuming substantial water during hydration; (2) This water depletion creates conditions thermodynamically favorable for the conversion of high-water-content phases (tobermorite) to their low-water-content counterparts (xonotlite) [40,41,42]. Notably, all samples in this study underwent standard curing before autoclave curing. Since LS contains substantial amounts of SO_3_ and carbonates, a significant amount of ettringite forms during the early 1-day stage, consuming a large portion of the free water in the cement paste. This further facilitates the formation of low-water-content xonotlite in the autoclaved L30.

Compared to standard curing for 28 days, autoclave curing for 3 h resulted in the formation of more CH and less C−S−H, indicating a lower degree of hydration in the cement paste at this stage. Larger crystals of CH tended to grow near the ITZ, which is detrimental to the durability of concrete [74,75]. However, the incorporation of SCMs helped reduce the CH content in the autoclave-cured cement paste. As shown in Table 4, the CH content of Ref, G30, S30, F30, and L30 decreased in order, indicating that the pozzolanic effect between LS and cement was the most pronounced under autoclave curing conditions. Although F30 also exhibited a lower CH content, it formed less C−S−H, which may limit its contribution to the overall strength development.

Although the activity of SS is lower than that of GBFS, the CH content in S30 is slightly lower than that in G30. This may be related to the finer particle size of GBFS (Figure 2). Under autoclave curing conditions, in addition to the pozzolanic reaction, GBFS can also act as a nucleation site to promote cement hydration, leading to the formation of more CH. Furthermore, the lower CH content in S30 is also associated with more severe carbonation, which converts more CH into CaCO_3_. This is evidenced by the higher CaCO_3_ content in S30, as shown in Table 4. These findings highlight the complex interactions between different SCMs and their influence on hydration products under autoclave curing conditions.

Under autoclave curing conditions, a significant amount of hydrogarnet is formed. Hydrogarnet is derived from hydrated aluminates, and its formation is accompanied by a reduction in solid-phase volume, which increases porosity and may lead to a decrease in strength. As indicated by the above analysis, the mass loss in the TG curve between 280~390 °C can, to some extent, reflect the amount of hydrogarnet formed. According to Table 4, the mass loss in the range of 280~390 °C for G30, L30, S30, Ref, and F30 decreases sequentially. This observed trend directly correlates with the Al/Si ratio in the raw materials—higher Al/Si ratios promote greater hydrogarnet formation in the composite cement paste. The underlying mechanism involves the Al/Si ratio’s critical role in phase selection: reduced ratios favor C_2_ASH_8_ phase stabilization while suppressing the formation of CAH_10_, C_2_AH_8_ and C_4_AH_13_ phases, consequently preventing their subsequent conversion to hydrogarnet [76]. However, although the Al/Si ratio in SS is higher than that in LS, the formation of hydrogarnet in L30 is relatively greater. This is primarily because the formation of hydrogarnet is not only related to the Al/Si ratio but also to the Al_2_O_3_ content in the raw materials, and the Al_2_O_3_ content in LS is significantly higher than that in SS. Therefore, controlling the Al/Si ratio in raw materials, especially the ratio of reactive Al_2_O_3_ to reactive SiO_2_, can help regulate the formation of hydrogarnet under high-temperature or autoclave curing conditions, thereby improving the long-term strength of autoclave-cured components.

In addition to hydrogarnet, hydrotalcite is also formed under autoclave curing conditions, which mainly depends on the reactive MgO content in the raw materials. However, due to the low MgO content in LS, no hydrotalcite is observed in L30. Although the MgO content in FNS is significantly higher than that in other SCMs, the MgO in FNS mainly exists in the form of forsterite and enstatite, and the reactive MgO content in the amorphous phase is limited. Therefore, the formation of hydrotalcite is also minimal.

Owing to the elevated temperatures associated with autoclave curing conditions, both ettringite and AFm phases within the cement paste undergo thermal decomposition, which leads to the formation of substantial quantities of gypsum or syngenite. The presence of these reaction products may trigger the risk of delayed ettringite formation (DEF), thereby compromising the volume stability of the resulting concrete [56,57]. Consequently, strict control of raw material SO_3_ content and rigorous regulation of the autoclave curing regime are imperative for autoclave-cured concrete products. These measures serve to minimize the content of ettringite and analogous phases generated during pre-curing, ultimately improving the long-term durability of the concrete.

#### 2.7.2. The Reactivity of SCMs

(1) Under standard curing conditions

Under standard curing conditions, the reactivity of GBFS, LS, SS, and FNS decreases sequentially (Figure 13). The reactivity of SCMs is primarily governed by their mineral and chemical compositions, in addition to particle fineness and specific surface area [77]. Among these parameters, the amorphous glass phase content in SCMs is the key determinant of their pozzolanic activity [78]. This phase possesses an unstable structure, rendering it more susceptible to dissolution in alkaline environments and subsequent participation in pozzolanic reactions. A notable example from this study is GBFS, whose mineral composition is dominated by an amorphous glass phase and thus exhibits significantly higher reactivity than FNS, which has a crystal phase-dominated matrix.

Furthermore, the content of reactive mineral phases (e.g., silicate and aluminate phases) in SCMs also modulates their reactivity. Such phases can directly participate in hydration reactions or accelerate cement hydration, thereby yielding more hydration products and improving the strength of the cementitious system. For instance, SS contains phases including C_2_S, C_2_F, and C_12_A_7_, which can directly participate in chemical reactions and contribute to strength development.

Meanwhile, the alkali (e.g., Na_2_O, K_2_O) and SO_3_ contents in SCMs also regulate the formation of hydration products [79]. Lithium slag serves as a typical case: it contains alkaline species such as sodium carbonate and sodium sulfate, coupled with a relatively high gypsum content, and thus maintains high reactivity despite a low amorphous glass phase content.

Therefore, the reactivity of SCMs is a comprehensive outcome of the synergistic effects of multiple factors, namely specific surface area, amorphous glass phase content, reactive mineral phase content, alkali content, and SO_3_ content.

(2) Under autoclave curing conditions

Under autoclave curing conditions, most components in cement and SCMs can participate in cement hydration, including spodumene and quartz in LS, as well as *f*-CaO and *f*-MgO in SS. However, under these conditions, forsterite ((Mg, Fe)_2_SiO_4_) and enstatite (MgSiO_3_) in FNS, which contain a high amount of SiO_2_, remain relatively stable. This stability limits the reactive content of FNS and consequently affects its activity under autoclave curing conditions.

The activity of SCMs under autoclave curing conditions primarily depends on their reactive SiO_2_ and Al_2_O_3_ content. As shown in Table 1 and the preceding analysis, LS has the highest content of reactive SiO_2_ and Al_2_O_3_, enabling it to achieve an activity level comparable to that of GBFS under autoclave conditions. This makes LS a viable alternative to GBFS as a new type of SCM. However, the activity of LS is still slightly lower than that of GBFS, mainly because LS is an acidic SCM that lacks self-hardening capability, and its reaction degree is significantly influenced by the alkalinity of the cement paste. As a result, when the LS content increases, the alkalinity of the blended cement paste decreases, which will in turn lead to a reduction in the proportion of LS that actually participates in the cement hydration reaction. This is evidenced by the presence of unreacted spodumene in the autoclave-cured L30 sample, as shown in Figure 4c. In contrast, GBFS, as an alkaline SCM primarily composed of an amorphous phase, not only participates in the pozzolanic reaction but also possesses self-hardening capability. Consequently, high-volume (50–70%) GBFS-based concrete has been widely used in practical engineering projects.

Additionally, the CaO content in SCMs is another critical factor, playing a key role in the formation of C−S−H. As shown in Table 1, the CaO content in LS is extremely low, while it is relatively high in GBFS. Although LS incorporation can significantly reduce the CH content, it also lowers the CaO content in cement-based materials, thereby limiting the extensive formation of C−S−H. Therefore, LS is more suitable for use in cement and concrete at low incorporation levels. To further investigate this, the authors replaced 20% of cement with LS and GBFS, respectively, and studied the mechanical properties of LS-blended cement mortar and GBFS-blended cement mortar under 80 °C steam curing for 7 days [37,43]. The results showed that the mechanical properties of LS-blended cement mortar were significantly superior to those of GBFS-blended cement mortar, both in terms of compressive strength and flexural strength. This further demonstrates that LS is more suitable for use at low incorporation levels. If high incorporation levels of LS are required, supplementary calcium and alkali must be added to enhance its reaction degree. However, at elevated LS incorporation levels, special attention should be paid to: (1) the relatively high gypsum content in LS that may induce internal sulfate attack in concrete [80], and (2) the elevated aluminum content which could promote the formation of additional hydrogarnet under high-temperature and/or high-pressure conditions, potentially leading to strength retrogression of concrete.

Under standard curing conditions, the mechanical performance of F30 is slightly inferior to that of S30. However, under autoclave curing conditions, F30 exhibits superior mechanical performance. This can be attributed not only to the formation of more hydration products in the FNS cement paste under autoclave curing conditions (Table 4) but also to the significant retardation of cement setting time caused by the incorporation of steel slag (Table 3). The retarding effect of steel slag on cement hydration partially offsets the promoting effect of autoclave curing on cement hydration. Therefore, compared to other SCMs, steel slag is not suitable for use in concrete prepared under autoclave curing.

#### 2.7.3. Future Investigations

The formation of certain hydration products, such as hydrogarnet and gypsum, can adversely affect concrete durability. Hence, it is essential to systematically investigate the temporal evolution of hydration processes, products, microstructure, and macroscopic properties in autoclaved specimens containing different SCMs. In addition, research should be directed toward developing effective methods to mitigate these adverse effects, such as by optimizing the curing regime and the type/content of SCMs.

Furthermore, among the four SCMs evaluated, lithium slag exhibits the lowest Ca/Si ratio, the highest Al content, and contains potentially reactive lithium salts. These characteristics suggest it holds promise for mitigating ASR [21,22,25,81]. Notably, the reactivity of its SiO_2_ and Al_2_O_3_ is significantly enhanced under high-temperature curing. Consequently, further investigation will focus on evaluating its durability performance as an SCM under elevated service temperatures, with particular emphasis on ASR suppression.

## 3. Conclusions

This study systematically examined the influence of ferronickel slag (FNS), lithium slag (LS), steel slag (SS), and ground granulated blast furnace slag (GBFS) on the early-age hydration products and mechanical properties of cementitious systems under autoclave curing at replacement levels of 0% and 30%, with comparative analysis against conventional 28-day standard curing conditions. Key results indicate that:

(1) LS incorporation facilitated the extensive formation of ettringite, endowing the cementitious system with superior flexural strength development compared with systems incorporating FNS and SS. However, a considerable proportion of Al_2_O_3_ and SiO_2_ in LS were encapsulated within inert crystalline phases, which restricted its pozzolanic reactivity under standard curing conditions and resulted in mechanical performance inferior to that of GBFS.
(2) SCMs facilitated the formation of low Ca/Si ratio C−S−H gels with spherical morphology and enhanced Al incorporation under standard curing conditions.
(3) Autoclaving significantly enhanced the reactivity of all SCMs, as evidenced by TG/DTG analysis. Compared to plain cement, the mass loss below 390 °C varied from 87.2% (FNS) to 106.5% (GBFS), while the content below 200 °C ranged between 87.0% (SS) and 104.6% (GBFS). Notably, CH content decreased substantially, with reductions to 70.1% (FNS), 47.7% (LS), 77.9% (SS), and 82.4% (GBFS) of the control values.
(4) Among all SCMs, autoclave curing exhibited the most significant activating effect on LS, which consequently demonstrated the greatest potential as a GBFS alternative in autoclaved concrete products. However, the low CaO content and acidic nature of LS inherently limit its use to low replacement levels unless supplementary alkalinity and CaO sources are incorporated.
(5) Compared to standard 28-day curing, autoclave curing resulted in a lower overall yield of hydration products in the cementitious systems and exhibited a marked tendency for CH formation. It also accelerated the crystallization of gypsum, hydrogarnet, and CASH phases, which are detrimental to the long-term performance of concrete.
(6) Autoclave curing transformed the calcium carbonate morphology in cementitious materials from the cubic or fibrous forms of standard curing into plate-like or polyhedral configurations.

## 4. Materials and Methods

### 4.1. Materials

The cement used in this study was P·II 52.5 Portland cement (PC). Ferronickel slag (FNS) powder, lithium slag (LS) powder, steel slag (SS) powder and ground granulated blast furnace slag (GBFS) were supplied by Jiangsu Rongda New Materials Co., Ltd. (Nantong, China). In this study, LS is a solid by-product obtained from lithium carbonate production via the sulfuric acid method. Its production involves key steps such as spodumene calcination, sulfuric acid roasting, leaching, neutralization, solid–liquid separation, and final carbonate precipitation [82]. The steel slag used in this study had undergone digestion and aging treatments.

ISO standard sand and tap water were used to prepare cement paste and mortar.

### 4.2. Sample Preparation and Test Methods

#### 4.2.1. Sample Preparation

Cement pastes were prepared with a water-to-cement ratio of 0.3, incorporating 30% replacement of cement by FNS, LS, SS, or GBFS. Five distinct paste formulations were developed: a reference plain cement paste (Ref) along with four composite pastes containing FNS (F30), LS (L30), SS (S30), and GBFS (G30) as partial cement replacements. After an initial 24 h standard curing period at controlled conditions of 20 ± 2 °C and 90 ± 5% relative humidity, the specimens were divided for subsequent curing protocols. One set remained under standard curing conditions for 28 days, while another set received autoclave treatment simulating typical aerated concrete production conditions. The autoclave regime involved exposure to 0.8 MPa steam pressure at 180 °C, achieved through a 3.5 h pressurization phase followed by a 3 h isothermal holding period. This curing regime is relatively consistent with those reported in the literature [1,11].

Complementary mortar specimens with dimensions of 40 × 40 × 160 mm were prepared to assess mechanical performance, employing identical curing schedules as the paste specimens. All mortar mixtures were prepared with a fixed mass ratio of 1:3:0.5 (binder: standard sand: water) to ensure uniform hydration conditions and enable consistent mechanical property evaluation across material systems.

#### 4.2.2. Test Methods

To comprehensively investigate the effects of SCMs on autoclaved cement-based materials, a series of standardized tests were conducted following Chinese national standards GB/T 1346-2024 [32], GB/T 2419-2005 [83], and GB/T 17671-2021 [84]. These tests evaluated key properties including water demand, setting time, soundness of cement pastes, as well as fluidity and compressive strength of cement mortars. The detailed test procedures are as follows:

Water demand: A standard cement paste was prepared by mixing 500 g of blended cement with water at a predetermined water-to-cement (*w*/*c*) ratio. The standard consistency water demand was determined using a Vicat apparatus (Shanghai Rongjida Instrument Technology Co., Ltd., Shanghai, China). It was defined as the *w*/*c* ratio at which the Vicat plunger penetrated to a point 5 mm to 7 mm from the bottom of the mold.

Setting time: Cement pastes were prepared at their standard consistency water demand. Initial and final setting times were determined using a Vicat apparatus. The initial setting time was recorded as the time elapsed from test commencement until the Vicat needle penetrated to a depth of 3–5 mm above the mold bottom. Subsequently, the mold was inverted, the initial needle was replaced with the final setting needle, and testing continued. The final setting time was defined as the cumulative time when the needle left only a circular indentation on the paste surface.

Soundness: The volume soundness of cement was tested using the Le Chatelier method. A cement paste of standard consistency was cast into a Le Chatelier mold equipped with dual indicator needles. After curing in a standard cabinet for 24 h, the initial distance between the needle tips was recorded. The mold and specimen were then boiled in a test chamber for 3 h. Once cooled to room temperature, the distance between the needle tips was measured again. The cement was considered to have passed the volume soundness test if the average increase in needle tip distance for two parallel specimens after boiling did not exceed 5 mm.

Fluidity of mortar: The mortar’s fluidity was determined using the flow table method. After subjecting the mortar specimen to 25 drops on a standard flow table, the spread diameters were measured in two perpendicular directions. The average of these two measurements was recorded as the mortar’s fluidity index.

Mechanical property: The flexural and compressive strengths of 40 × 40 × 160 mm prismatic mortar specimens were determined in accordance with Chinese National Standard GB/T 17671-2021 using a YAW-300C integrated cement flexural and compressive testing machine (Zhongluchang Instrument and Equipment Manufacturing Co., Ltd., Jinan, China). The loading rates were set at 50 ± 10 N/s for the flexural strength test and 2400 ± 200 N/s for the compressive strength test.

The microstructural characteristics and phase composition of hydration products formed under autoclave curing conditions were systematically analyzed through multiple analytical techniques. X-ray diffraction (XRD) analysis was performed using a D8 Discover diffractometer (Karlsruhe, Germany) with CuKα1 radiation, operating at 40 kV and 36 mA with a scanning rate of 3°/min. Microstructural examination was conducted using a Sirion field-emission scanning electron microscope (FEI, Hillsboro, OR, USA)) equipped with energy-dispersive X-ray spectroscopy (EDS), with samples prepared by fracturing fresh cement paste and coating with gold sputtering.

Thermogravimetric (TG/DTG) analysis was employed to quantify the non-evaporable water and calcium hydroxide (CH) content in the hydration products. The measurements were carried out using an STA 449 F3 Jupiter thermogravimetric analyzer (Selb, Germany) under nitrogen atmosphere, with a controlled heating rate of 10 °C/min from ambient temperature to 950 °C. This comprehensive analytical approach enabled detailed characterization of the phase evolution and microstructural development in the autoclaved cement systems containing different SCMs.

## Figures and Tables

**Table 1:** The chemical compositions of PC, FNS, LS, SS and GBFS, wt.%.

Materials	CaO	SiO_2_	Al_2_O_3_	SO_3_	Fe_2_O_3_	MgO	Na_2_O	K_2_O	Cr
PC	64.47	20.87	4.87	2.52	3.59	2.13	0.11	0.65	0.04
FNS	11.49	47.61	6.56	0.52	13.24	15.94	0.80	0.18	0.70
LS	4.53	62.40	22.10	6.73	1.06	0.49	0.89	0.52	-
SS	38.62	18.46	7.12	1.08	22.5	6.60	0.21	0.15	-
GBFS	36.05	34.67	16.52	2.53	0.29	3.90	-	0.33	-

**Table 2:** Mineral composition, loss on ignition and density of raw materials.

Materials	Mineral Composition	LOI (%)	Density (g/cm^3^)
PC	C_3_S, C_2_S, C_3_A, C_4_AF, CaCO_3_, CaSO_4_·2H_2_O	2.4	3.05
FNS	(Mg, Fe)_2_SiO_4_, MgSiO_3_	1.4	2.99
LS	LiAlSi_2_O_6_, CaSO_4_·2H_2_O, SiO_2_	5.7	2.60
SS	C_2_S, C_2_F, C_12_A_7_, RO phase, Ca(OH)_2_, CaCO_3_, *f*-CaO, *f*-MgO	6.2	3.27
GBFS	Amorphous phase	2.0	2.90

**Table 3:** Physical properties of blended cement pastes and mortars.

Samples	Water Demand (wt.%)	Setting Time (h: min)		Le Chatelier Soundness (mm)	Flow of Mortar (mm)
		Initial	Final		
Ref	27.4	2:52	3:57	0.5	210
F30	27.4	4:10	5:25	0.5	210
L30	28.9	3:30	4:40	0.5	190
S30	27.2	4:40	6:25	3.0	220
G30	28.2	3:45	5:00	0.5	200

**Table 4:** Mass loss at a specific temperature range, CH and CaCO_3_ content in various cement pastes, wt.%.

Curing Condition	Samples	Mass Loss	<390 °C	<200 °C	280~390 °C	CH	CaCO_3_
Standard curing	Ref	20.62	11.04	7.18	2.10	20.00	6.77
Autoclave curing	Ref	19.96	8.65	4.15	2.99	26.39	5.65
	F30	18.66	7.54	3.72	2.39	18.50	3.68
	L30	16.71	9.07	4.16	3.17	12.58	4.70
	S30	18.41	8.28	3.61	3.05	20.55	8.36
	G30	18.67	9.21	4.34	3.26	21.74	4.45

**Table 5:** Chemical compositions of the phases in areas 1~7 of Figure 7, at.%.

Phases	C	O	Si	Ca	Al	S	Fe	Mg	K
1		38.38	15.13	40.95	2.07	0.99	1.82		0.66
2		60.87	22.96	12.04			0.94	2.43	0.76
3		62.19	11.13	18.39	4.29	1.45	1.27	0.70	0.57
4	14.21	66.69	5.81	8.39	1.60	0.27	0.61	1.49	0.94
5	20.45	52.87	8.75	12.56	1.95		0.55	2.48	0.38
6	38.50	37.95	1.48	21.33					0.73
7	32.56	43.80	5.59	18.05					

## Data Availability

All data generated or analyzed during this study are included in this submitted article.

## Abbreviations

The following abbreviations are used in this manuscript:

SCMs	Supplementary cementitious materials
FNS	Ferronickel slag
LS	Lithium slag
SS	Steel slag
GBFS	Ground granulated blast-furnace slag
ASR	Alkali-silica reaction
UHPC	Ultra-high performance concrete
*f*-CaO	Free calcium oxide
*f*-MgO	Free magnesium oxide
C−S−H	Calcium silicate hydrate
DEF	Delayed ettringite formation
PC	Portland cement
XRF	X-ray fluorescence
LOI	Loss on ignition
XRD	X-ray diffractometer
SEM-EDS	Scanning electron microscope coupled with an X-ray energy dispersive spectrometer
TG-DTG	Thermogravimetric and derivative thermogravimetric analyses
CH	Calcium hydroxide
AFt	Ettringite
Mc	Monocarbonate
Hc	Hemicarbonate
AFm	Monosulfoaluminate hydrate
LDH	Layered double hydroxide
ITZ	Interfacial transition zone
CASH	Calcium aluminosilicate hydrate
